# Amyloid **β**-Related Angiitis Causing Coma Responsive to Immunosuppression

**DOI:** 10.1155/2012/678746

**Published:** 2012-08-23

**Authors:** Shennan A. Weiss, David Pisapia, Stephan A. Mayer, Joshua Z. Willey, Kiwon Lee

**Affiliations:** ^1^Department of Neurology, Columbia University, 710 W. 168th Street, New York, NY 10032, USA; ^2^Department of Pathology, Columbia University, 630 W. 168th Street, New York, NY 10032, USA

## Abstract

*Introduction*. Amyloid-beta-related angiitis (ABRA) is a form of CNS vasculitis in which perivascular beta-amyloid in the intracerebral vessels is thought to act as a trigger for inflammation mediated by CD68+ macrophages and CD3+ T lymphocytes. Patients with severe ABRA may develop coma responsive to immunosuppressive treatment. *Case Presentation*. A 57-year-old man presented to the neurological intensive care unit febrile, obtunded, and with a left hemiparesis. He had suffered from intermittent left arm weakness and numbness for several months prior. Serum and cerebrospinal fluid studies showed a lymphocytic leukocytosis in the cerebrospinal fluid (CSF), but no other evidence of infection, and the patient underwent a brain biopsy. Histopathological examination demonstrated amyloid angiopathy, with an extensive perivascular lymphocytic infiltrate, indicative of ABRA. The patient was started on cyclophosphamide and steroids. Following a week of treatment he awakened and over several weeks made a significant neurological recovery. *Conclusions*. ABRA can have a variety of clinical presentations, including impairments in consciousness and coma. Accurate pathological diagnosis, followed by aggressive immunosuppression, can lead to impressive neurological improvements. This diagnosis should be considered in patients with paroxysmal recurrent neurological symptoms and an accelerated progression.

## 1. Introduction 

Deposition of beta-amyloid in the cerebral vasculature affects 30% of the healthy elderly and 90% of those with Alzheimer's disease [1]. This process has been termed amyloid angiopathy and is a recognized cause of cerebral microhemorrhages and cerebral lobar hemorrhages. Amyloid-beta-related angiitis (ABRA) is a rare complications of amyloid angiopathy and is considered a form of CNS angiitis in which perivascular of beta-amyloid is thought to act as a trigger for inflammation [2]. Primary CNS vasculitis, encompassing all subtypes including ABRA, is rare occurring in 2.4 cases per 1,000,000 patient years [3]. We describe a patient with ABRA who was comatosed and responded to aggressive immunosuppression. 

## 2. Case Presentation

**Figure 1:**
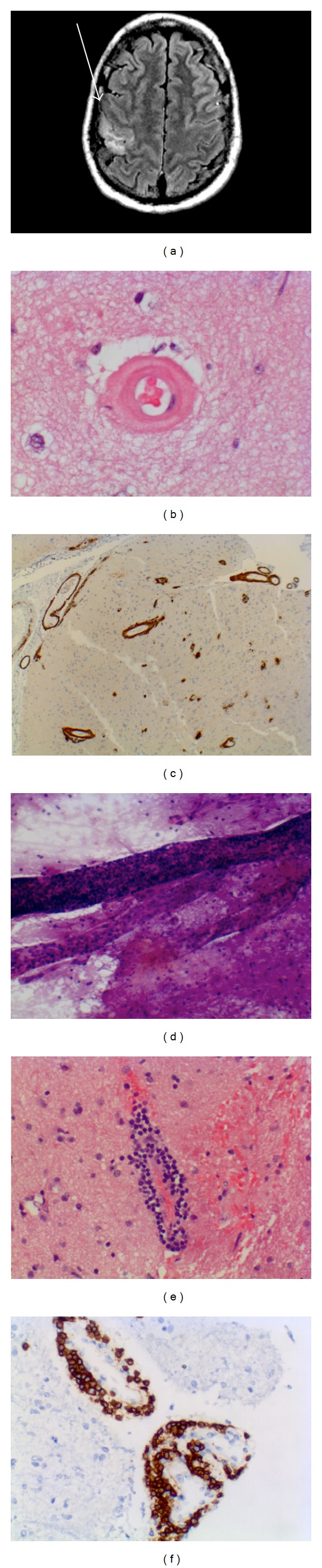
Amyloid-beta-related angiitis. (a) Magnetic resonance T2 FLAIR imaging at admission to Neuro ICU note hyperintense lesion in the grey and white matter in the right sensorimotor cortex. (b) Hematoxylin-eosin-stained section shows characteristic double barrel lumen appearance of an amyloid laden vessel. (c) Immunohistochemistry using monoclonal antibody against beta-amyloid reacting to vessels in the meninges and parenchyma. (d) Squash prep at time of surgery-blood vessel and extensive perivascular lymphocytic infiltrate. (e) Lymphocytic response with intramural vascular inflammation. (f) Antibody against CD3 demonstrates a perivascular T-cell infiltrate.

A 57-year-old man with a past history of hypertension, diabetes mellitus type 2, hyperlipidemia, and crack cocaine use was admitted to a community hospital for flu like symptoms of one-week duration and ongoing paroxysmal episodes of left hand numbness and weakness occurring over several months. In the emergency department patient was febrile to 38.9°C. He had a slight peripheral white count and CSF demonstrated a lymphocytic pleocytosis (190 white blood cells (WBC), 81% lymphocytes), with normal protein and glucose. He was started on acyclovir, ceftriaxone, ampicillin, vancomycin, and 3 days of methylprednisolone. Over the course of eight days the developed worsening left arm weakness, dysarthria, confusion, agitation, and episodic right eye gaze deviation. Upon arrival to the intensive care unit his examination was notable for fever, tachycardia and not opening his eyes to voice or noxious stimuli. He exhibited roving spontaneous eye movements, with present oculo-cephalic, corneal, and gag reflexes. He had normal tone in all limbs and left-sided hemiplegia. A repeat lumbar puncture demonstrated 85 red blood cells (RBC), 47 WBC (94% lymphocytes), a protein of 61, and glucose of 71 and opening pressure of 250 mm H_2_O. Magnetic resonance imaging with contrast revealed several T2 signal abnormalities in the deep cerebellar white matter, right posterior thalamus, and right posterior frontal gyri (Figure 1(a)). No corresponding regions with an increased apparent diffusion coefficient (ADC) were identified. The lesions did not enhance with gadolinium and gradient phase echo showed no evidence of blood products. Cerebral angiography demonstrated no obvious abnormalities. Continuous EEG showed no seizures. He was continued on acyclovir, ceftriaxone, vancomycin, ampicillin, as well as steroids. CSF viral, bacterial, and fungal cultures were normal, as was a paraneoplastic panel. The patient's exam was unchanged for the first three days, he exhibited autonomic instability requiring either norepinephrine or nicardipine drips and was cooled to achieve normothermia. The ANA test was negative and the patient did not harbor ANCA antibodies, mycoplasma IgM, or cryoglobulins. The patient underwent a stereotactic right parietal craniotomy for biopsy of a cortical region with T2 signal abnormalities, as well as surrounding dura mater. The biopsy showed evidence of ABRA (Figure 1(a)).

Hematoxylin and eosin stained sections showed an intramural and perivascular inflammatory infiltrate composed predominantly of mature appearing T cells. Many of the small to medium caliber vessels showed markedly thickened, rigid appearing walls with deposition of a glassy, hypereosinophilic material that stained strongly with an immunostain for beta-amyloid. Occasional vessels showed formation of concentric double rings of the hypereosinophilic material within the vessel wall. Larger vessels within the leptomeninges also showed beta amyloid deposition. A trichrome stain also showed a mild to moderate degree of collagen deposition in these same vessels. No granulomatous inflammation was identified. Staining for CD20 showed only rare B cells in the inflammatory infiltrate. The cerebral cortex appeared hypercellular secondary to the presence of a mixed inflammatory infiltrate comprising macrophages/microglia (highlighted by staining for CD68) and mature appearing CD3-positive T cells as well as a reactive astrogliosis (highlighted by staining for GFAP). Additionally, several microscopic subacute infarctions were identified each with a robust histiocytic response. Beta amyloid monoclonal antibody immunohistochemistry revealed both amyloid angiopathy and diffuse-type plaques within the cortical neuropil. Immunostaining for tau protein was negative for neurofibrillary tangles except for rare neurites within an immature plaque. 

Six days after initiating high-dose methylprednisolone the patient's exam improved with evidence of visual tracking and following simple commands using his right hand. He was subsequently started on a course of cyclophosphamide 0.75 mg/m^2^ body surface area (BSA) and his examination gradually improved. At discharge to an acute rehabilitation facility eight days later the patient was mildly lethargic, with a residual left-sided weakness and improving dysarthria. An MRI at discharge demonstrated reduced FLAIR hyperintense signal, as well as a left cerebellar microhemorrhage. The patient's serum was sent for ApoE genotyping, and he was found to be a E2/E3 heterozygote. 

## 3. Discussion

Case series of patients with ABRA have demonstrated that the most common clinical feature is mental status changes. Common CSF findings include an elevated protein and a lymphocytic pleocytosis [2]. MRI often demonstrates T2 hyperintense lesions extending through the cortical white matter and often grey matter, suggestive of breakdown of the blood-brain barrier and a reversible leukoencephalopathy [4]. Cerebral angiography is suggestive of vasculitis in a minority of patients, perhaps due to involvement of exclusively medium- and small-sized vessels. In every case on brain biopsy microglia, macrophages, and T cells surround amyloid laden vessels [2]. CD4+ T cells may be indicative of an adaptive autoimmune response to beta-amyloid [5]. The predisposing factors for ABRA are not yet clear. In one small study, 75% of patients were APOE4 homozygotes. The Apo E2/E3 genotype was the second most common [6, 7]. ApoE genotype has been hypothesized to be critical in regulating vascular deposition of amyloid-beta [8, 9]. 

After initiating anti-inflammatory treatment consisting of steroids or cyclophosphamide for a duration ranging from two weeks to several months, a majority of patients with ABRA show improvement [7]. However, some patients relapse, and other patients do not improve or progressively decline. Typically the improvement occurs over the first five months after the initial episode. 

ABRA is an important clinical entity from the perspective of developing effective and safe immunotherapeutics for the treatment of Alzheimer's disease. In 2000 Elan therapeutics lead a trial in which 64 patients were immunized with an amyloid-beta 42 peptide. While many of these patients exhibited effective removal of plaque on autopsy, 6% developed an inflammatory complication leading to cessation of the trial [10]. It is thought that an inflammatory mechanism similar to ABRA mediated this response [2]. Another more recent trial involving bapineuzumab, a monoclonal antibody against amyloid-beta was found to result in asymptomatic cortical vasogenic edema, particularly in ApoE4 homozygotes, potentially due to inflammation [11]. 

## 4. Conclusion

ABRA is a rare clinical entity but should be considered in patients with recurrent intermittent neurological symptoms that rapidly progress. The severity of the clinical syndrome is variable but coma can result. Effective histopathological diagnosis is critical, since in many cases ABRA is reversible.
